# A novel MYH9 variant in a Chinese patient with MYH9 related disease

**DOI:** 10.1007/s00277-026-07181-z

**Published:** 2026-08-05

**Authors:** Haocheng Li, Xiang Li, Wanlei Sun, Shaoqin Liu, Mengyao Han, Jiayi Feng, Wenfeng He

**Affiliations:** 1 Queen Mary School, Nanchang University, Nanchang, 330006 China; 2 Department of Clinical Laboratory, The Second Affiliated Hospital, Jiangxi Medical College, Nanchang University, Nanchang, 330000 China; 3 Department of medical genetics, Molecular Medicine of Jiangxi Key Laboratory, The Second Affiliated Hospital, Jiangxi Medical College, Nanchang University, 330000 Nanchang, China

**Keywords:** MYH9-related disease, Macrothrombocytopenia, Mutation, c.5765 + 2T > G, Whole exome sequencing

## Abstract

To investigate the novel genetic mutations and clinical characteristics in a patient with MYH9 related disease in order to enhance understanding of the disease. Clinical information was obtained, including medical history, pertinent laboratory tests, and family history. Whole exome sequencing (WES) was conducted on the patient and her parents. A 19-year-old female presented with a history of epistaxis and chronic thrombocytopenia. Her aunt and brother also have a history of thrombocytopenia. Sequencing analysis revealed mutations of MYH9 gene (c.5765 + 2T > G) and HBB gene (c.126_129del) in the patient, with the c.5765 + 2T > G mutation being previously unreported. The splice site mutation of MYH9 was predicted to be pathogenic by Mutation Taster and Polyphen software programs. As a result of identifying this splicing site mutation in this particular case, we have added c.5765 + 2T > G to the broad spectrum of potential MYH9 mutations.

## Introduction

MYH9-related disorders (MYH9-RD) are a rare group of autosomal dominant genetic conditions characterized by thrombocytopenia, giant platelets, and inclusion bodies within neutrophils. Additional extra-haematological manifestations can also occur, which are mainly renal dysfunction, early-onset cataracts, and hearing impairment [1].

Based on the variability in clinical manifestations, MYH9-RD is classified into May-Hegglin anomaly (MHA), Fechtner syndrome (FTNS), Epstein syndrome (EPS), and Sebastian syndrome (SBS) [2]. The application of sequencing technology revealed that the aforementioned four diseases were caused by mutations in the MYH9 gene, which is situated on chromosome 22q11-13 and encodes the non-muscle myosin heavy chain IIA (NMMHC-IIA) [3, 4]. The Class IIA non-muscle myosin is a widely distributed hexameric motor protein composed of two heavy chains and two pairs of light chains. It is involved in various cellular processes, including cell spreading, motility, maintenance of cell morphology, and cytokinesis [5]. The disease has an estimated incidence of 1 in 100,000, with 20% to 40% of instances being sporadic [6]. Due to insufficient clinical awareness, many patients have been misdiagnosed with chronic immune thrombocytopenia and consequently subjected to inappropriate treatments such as immunosuppressive therapy and splenectomy [7].

Numerous studies have been published both nationally and internationally regarding MYH9-RD, yet the number of reported cases remains limited. This research identifies a novel mutation in the MYH9 gene associated with MYH9-related disease. The study aims to provide a comprehensive analysis of the clinical phenotypes and genetic mutation characteristics of MYH9-RD patients to advance the understanding of this condition.

### Case presentation

**Fig. 1:**
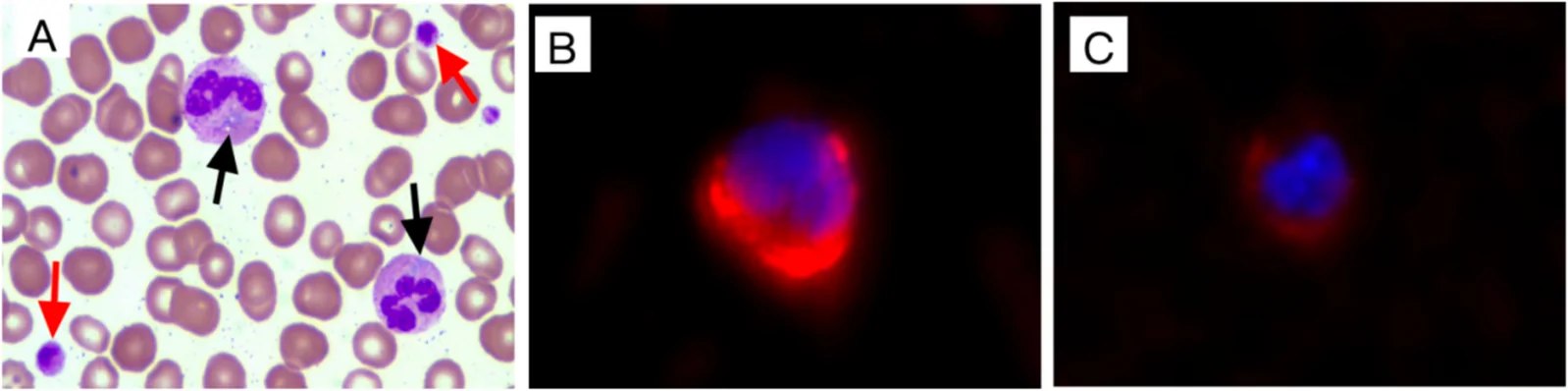
Peripheral blood smear and NMMHC-IIA immunofluorescence staining. (**A**) The giant platelets (red arrows) and basophilic inclusions in the neutrophils (black arrows) were observed in peripheral blood smear. (**B**) Abnormal aggregation of the MYH9 protein in the patients’ neutrophils using NMMHC-IIA immunofluorescence staining. (**C**) Normal MYH-9 distribution in control neutrophils

The patient was a 19-year-old Chinese woman with a 7-month history of thrombocytopenia. Seven months ago, during an examination for nasal bleeding, a platelet count of 25 × 10^9^/L was detected. Despite the finding, no treatment was administered. Subsequent routine blood tests consistently revealed low platelet counts ranging from 20 to 30 × 10^9^/L. In February 2022, the patient was admitted to our hematology department with a diagnosis of thrombocytopenia. Her aunt and cousin both have thrombocytopenia, and genetic sequencing revealed a MYH9 mutation (c.5765 + 2T > G). Physical examination revealed scattered petechiae and ecchymoses across the skin. No apparent abnormality was observed during the hearing and eye examinations. Routine blood tests showed hemoglobin (Hb) was 121 g/L, mean corpuscular volume (MCV) was 64.2 fL, mean corpuscular Hb was 19.3 pg, and reticulocyte count was 152.30 × 10^9^/L. Additionally, the platelet count was significantly reduced to 25 × 10^9^/L, which is significantly lower than the normal range of 100–300 × 10^9^/L. Platelet function tests showed significantly reduced maximum aggregation rates for AA and ADP, measuring 17.81% and 15.82%, respectively (normal range: 52–84%). Positive platelet autoantibodies, including anti-GPIIb and anti-GMP140 antibodies, were detected. The aspartate aminotransferase level was 39.83 U/L, no renal function abnormalities. Four coagulation tests, D-dimer, serum iron, ferritin, folic acid, and vitamin B12 levels were within normal ranges. Abdominal ultrasonography revealed splenomegaly. Transthoracic echocardiogram: mild regurgitation of mitral valve, tricuspid valve, aortic valve. Bone marrow aspiration indicated markedly active bone marrow hyperplasia, with a reduced granulocyte-to-erythrocyte ratio and significant hyperplasia in the granulocytic, erythroid, and megakaryocytic lineages, accompanied by maturation defects in the megakaryocytic lineage and thrombocytopenia. The peripheral blood smear showed giant platelets (Fig. 1A, red arrows) and basophilic inclusions in the neutrophils (Fig. 1A, black arrows) using the Wright-Giemsa stain. Subsequently, NMMHC-IIA immunofluorescence staining was performed on peripheral blood smears using the NMMHC-IIA rabbit polyclonal antibody(Affinity, Inc, China). The results revealed abnormal aggregation of the MYH9 protein in the patients’ neutrophils (Fig. 1B and C).

### WES and data analysis

**Fig. 2:**
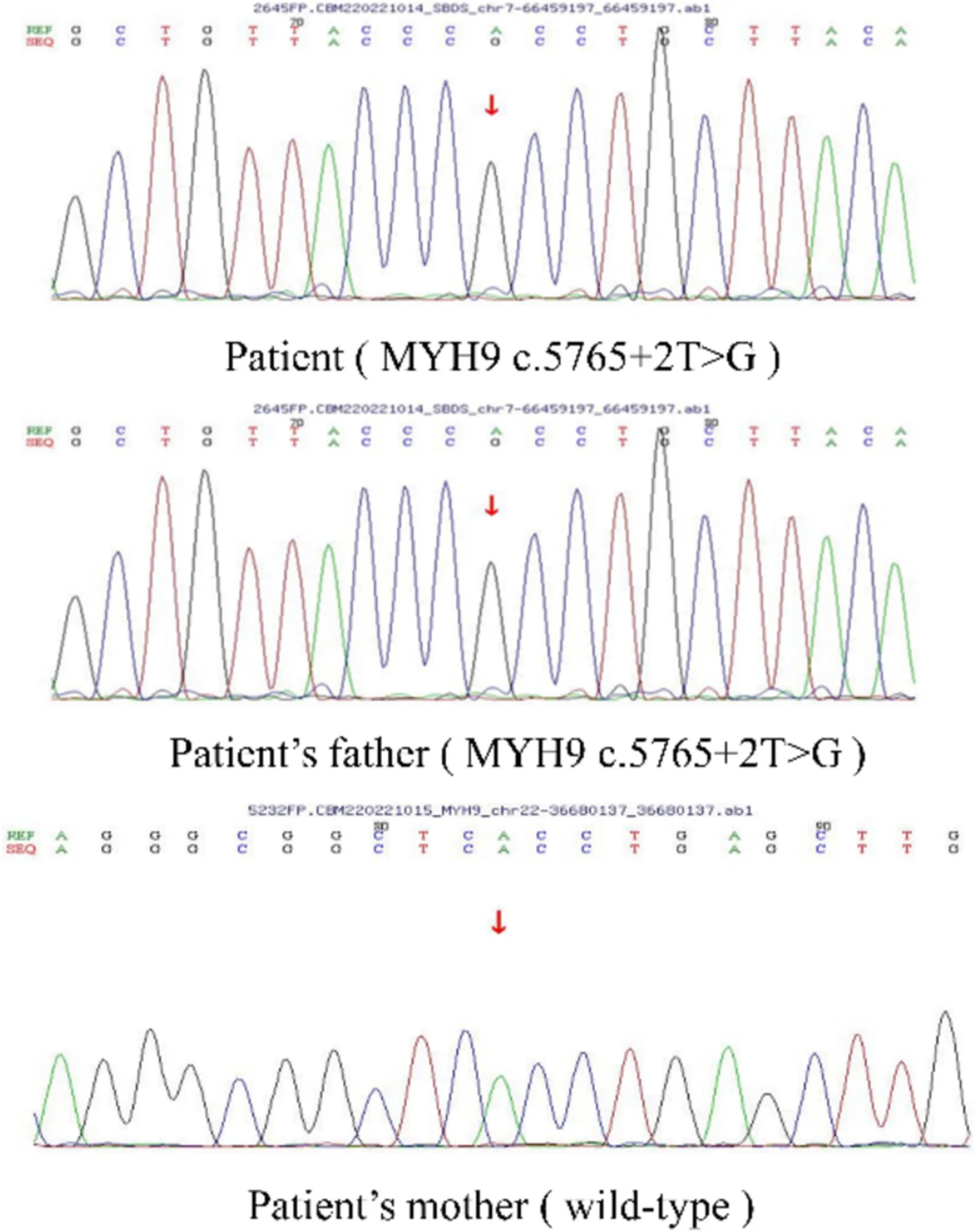
The c.5765 + 2T > G sequencing map of the MYH9 gene. The patient and her father carried the c.5765 + 2T > G variant; the patient’s mother was wild-type; the red arrow indicated the location of the mutation

**Fig. 3:**
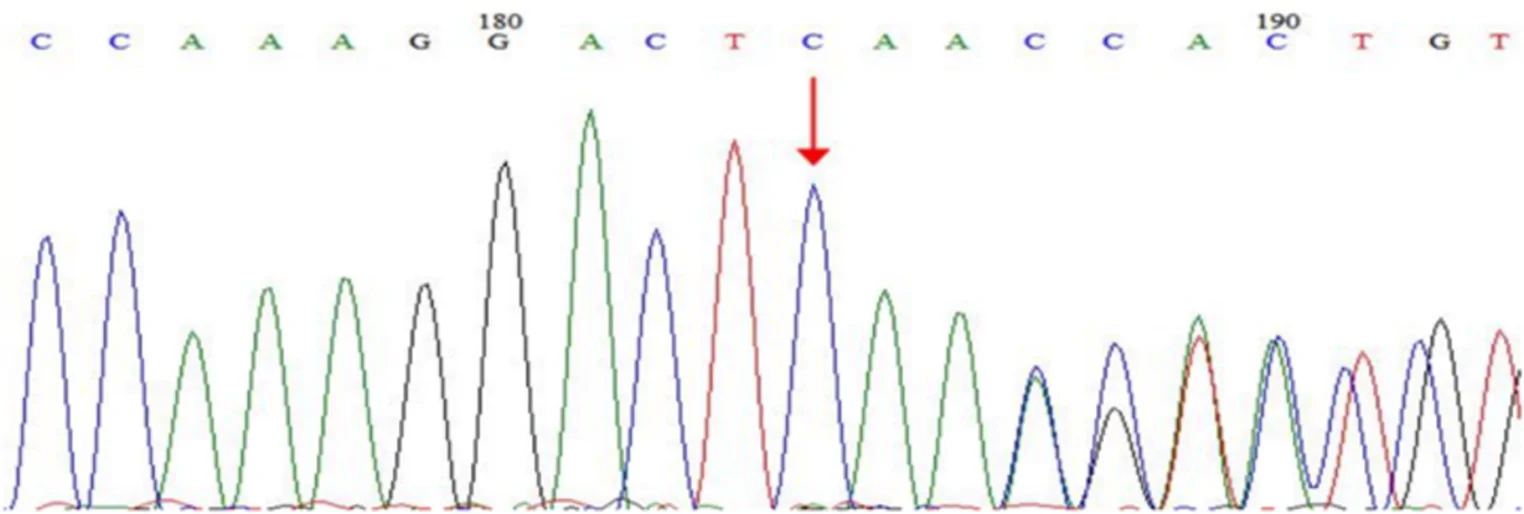
The c.126_129del sequencing map of the HBB gene

To make a precise diagnosis, we then performed whole-exome sequencing (WES) of peripheral venous blood specimens from the patient and her parents. The method is as follows: collect 2mL of peripheral blood from the patient, extract genomic DNA, prepare a DNA library using a hybrid capture method after fragmentation, adapter ligation, amplification, and purification, then detect the exonic regions and adjacent intronic regions (20 bp) of 20,099 genes in the human exome using a high-throughput sequencing platform. Compare the sequencing data with the human genome reference sequence hg19 (GRCh37) and evaluate the coverage and sequencing quality of the target regions accordingly. Assess the pathogenicity of variants according to the 2015 American College of Medical Genetics and Genomics (ACMG) guidelines for interpretation. WES identified a novel mutation in the MYH9 gene of the patient, specifically the c.5765 + 2T > G mutation in exon 40. This mutation was also present in the patient’s father but was absent in the patient’s mother (Fig. 2). Additionally, a heterozygous c.126_129del (p.F42fs) mutation in the HBB gene was detected in the patient (Fig. 3). We utilized PolyPhen-2 (http://genetics.bwh.harvard.edu/pph2/) to evaluate the functional impact of the newly identified MYH9 mutation on the protein sequence. To further assess the potential pathogenicity of this sequence variation, we employed Mutation Taster (http://www.mutationtaster.org), which systematically predicted whether the nucleotide alteration represents a deleterious mutation or a benign polymorphism. The splicing mutation of NM_002473 in exon 40 of MYH9 was predicted to be pathogenic by Mutation Taster, with a score of 0.9999965. The PolyPhen-2 model also predicted that this sequence variation would have a detrimental impact, with a score of 0.987 (sensitivity, 0.73; specificity, 0.96).

### Bioinformatics analysis

The protein-protein interaction (PPl) network associated with MYH9 was constructed using STRING (https://string.db.org/). The target genes in PPI network downloaded from STRING were used for Gene Ontology (GO) classification analysis.

**Fig. 4:**
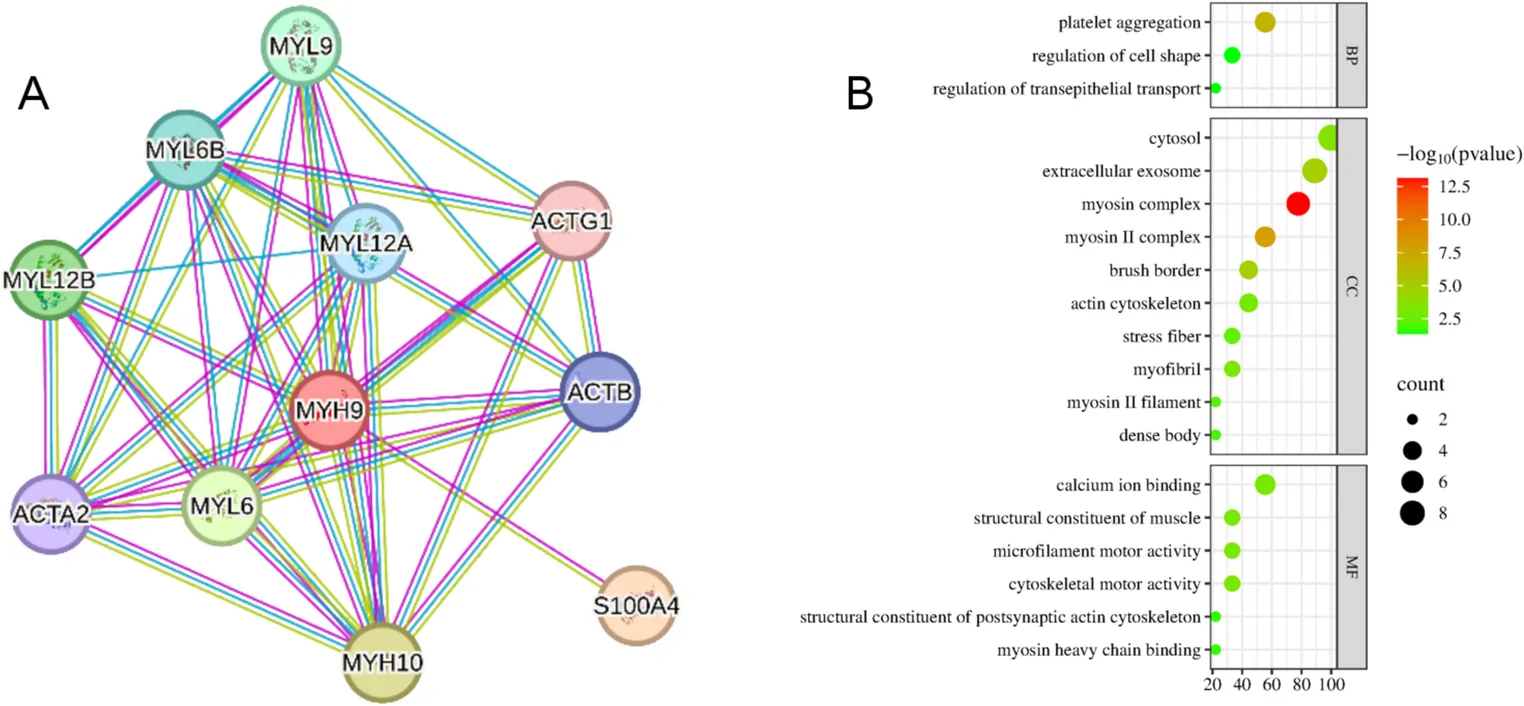
**A** The top 10 proteins interacting with MYH9 identified by PPI network analysis; **B** GO enrichment analysis of the top 10 proteins

The PPI network analysis of interacting genes with MYH9 was produced by STRING. As shown in Fig. 4A, the PPI network consisted of 11 nodes and 41 edges. The top three proteins with higher degrees are MYL6 (degree = 9), MYL6B (degree = 9) and MYL9 (degree = 9). The GO enrichment analysis showed that these genes were enriched in regulation of platelet aggregation and regulation of cell shape of BP, histone methyltransferase complex and methyltransferase complex of CC, histone-lysine N-methyltransferase activity and histone methyltransferase activity (H3-K4 specific) of MF (Fig. 4B).

## Discussion

MYH9-RD is a clinical condition that is frequently misdiagnosed as ITP because automated blood cell analyzers may miss a significant number of platelets and underestimate platelet counts [8]. Although the presence of Döhle-like inclusions in the cytoplasm of neutrophils on peripheral blood smears is a hallmark of MYH9-RD, the difficulty in detecting these inclusions can sometimes lead to misdiagnosis. Therefore, we consider immunofluorescence analysis or genetic testing as crucial methods for accurate diagnosis. Immunofluorescence staining enhances diagnostic sensitivity by allowing the detection of NMMHC-IIA inclusions that are not visible under light microscopy through specific antibody staining (anti-NMMHC-IIA) [9]. However, a definitive diagnosis relies on the detection of mutations in exon 41 of the MYH9 gene.

Using sequence analysis, we identified the c.5765 + 2T > G variant is a base substitution occurring at the splice site of exon 40 of the MYH9 gene. The WES from the patient’s family analysis revealed that the same mutation was also detected in the father’s peripheral blood samples, indicating that the mutation is inherited from the father. In theory, this mutation has the potential to disrupt mRNA splicing, thereby influencing the normal structure and function of the translated protein product. This mutation was anticipated to be detrimental by both the SIFT and Polygen-2 software. MYH9 is a large gene consisting of 41 exons, with six mutation hotspots located at residues 96 (exon 2), 702 (exon 17), 1165 (exon 27), 1424 (exon 31), 1841 (exon 39), and 1933 (exon 41) [10]. The coding regions from exons 2 to 20 encode the motor domain of NMMHC-IIA, while exons 21 to 41 encode the tail domain of the protein. Research has shown that the clinical manifestations of MYH9-RD patients were correlated with the mutation site. When mutations occurred in the motor domain of NMMHC-IIA, patients tended to experience more severe bleeding and were at a higher risk of developing significant renal dysfunction and deafness. In contrast, mutations in the tail domain were associated with a lower risk of bleeding and non-hematologic symptoms, with patients typically not developing renal dysfunction or cataracts. Hearing impairment was generally observed only in older age [11]. In our report, the newly splicing site mutation was located in the tail domain of NMMHC-IIA, and the patient exhibited a milder disease phenotype, which also corroborates previous observations.

Currently, there is no effective treatment for MYH9-RD. The primary therapeutic strategy involves the prevention of bleeding, the avoidance of drugs that impair platelet function, and the circumvention of invasive dental procedures. In emergency situations, platelet transfusions and artificial stimulation of platelet production may be administered to prevent hemorrhage [12]. Interventions such as immunosuppressants intravenous immunoglobulin, corticosteroids, and splenectomy have largely proven ineffective. Thrombopoietin receptor agonists (TPO-RAs), such as eltrombopag and romiplostim, have been shown to effectively increase platelet counts [13]. Additionally, renin-angiotensin system blockers, such as ramipril and telmisartan, have been shown to significantly reduce proteinuria in patients with this condition [14]. It is crucial for patients to undergo regular monitoring, including blood cell analysis, urinalysis, renal function tests, as well as ophthalmological and auditory examinations, with timely intervention as symptoms develop.

In conclusion, MYH9-RD is an uncommon autosomal dominant genetic condition that frequently goes unnoticed or is misidentified. It is beneficial to the early and accurate diagnosis of this condition to increase knowledge about it, particularly with regard to the clinicopathological signs of the disease in the liver, kidney, blood, ear, eye, and other organs. It is also important to pay attention to family history enquiries. Currently, there are limited targeted treatment options available, and additional research is required to ascertain the pathophysiology and therapeutic targets.

## Data Availability

All data are available on request from the authors.
